# Supplementary material for: Development of Photocrosslinking Probes Based on Huwentoxin-IV to Map the Site of Interaction on Nav1.7
Source: Cell Chem Biol. 2020 Mar 19;27(3):306–313.e4. doi: 10.1016/j.chembiol.2019.10.011 (PMC7083225; doi:10.1016/j.chembiol.2019.10.011)

Cell Chemical Biology, Volume *27*

Supplemental Information

Development of Photocrosslinking Probes Based on Huwentoxin-IV to Map the Site of Interaction on Nav1.7

Foteini Tzakoniati, Hui Xu, Tianbo Li, Natalie Garcia, Christine Kugel, Jian Payandeh, Christopher M. Koth, and Edward W. Tate

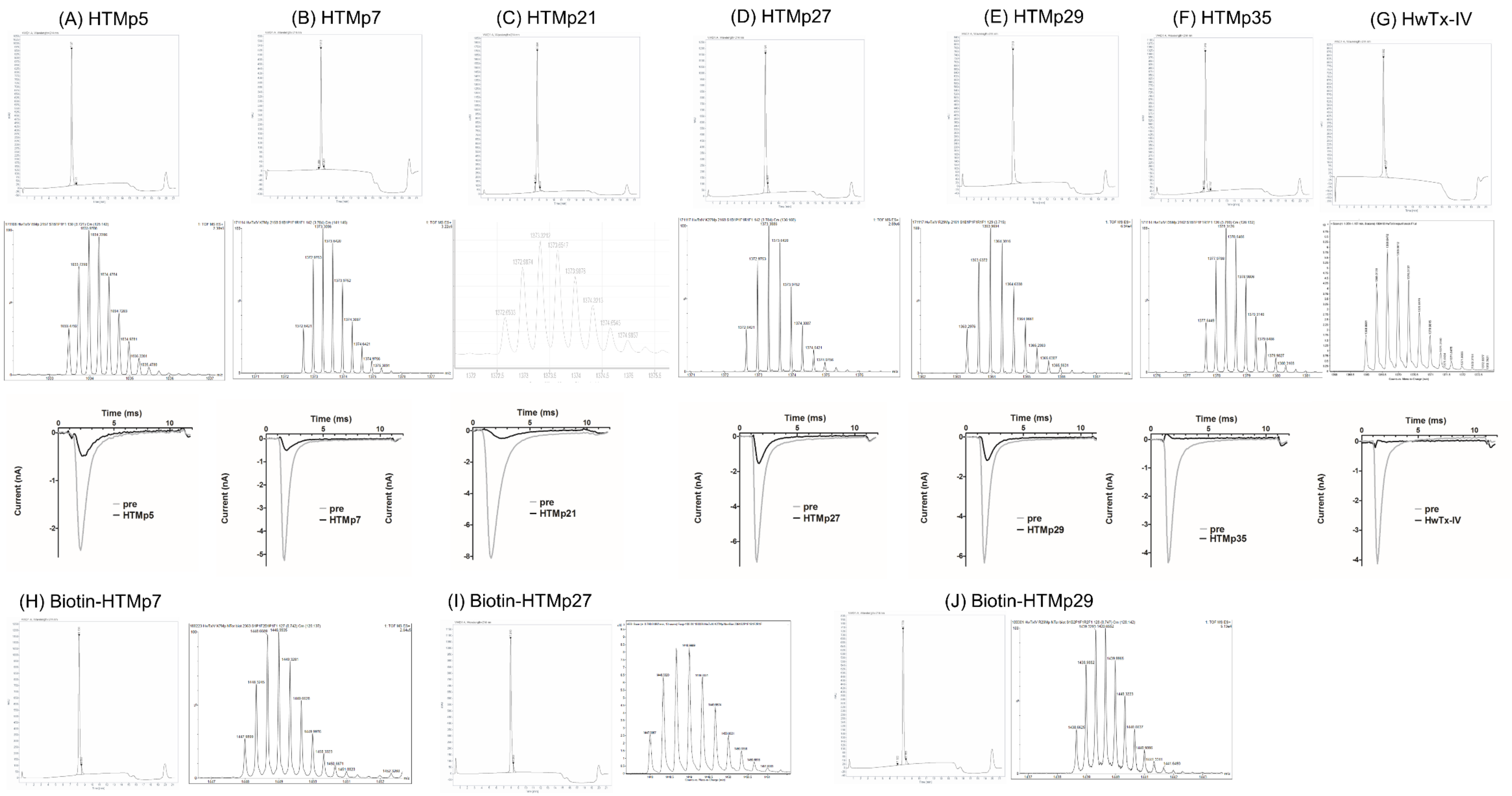

Figure S1. Related to Figure 1. LCMS traces of peptides and effects at 200 nM on CHO cells expressing Nav1.7

(A) HTMp5 (B) HTMp7 (C) HTMp21 (D) HTMp27 (E) HTMp29 (F) HTMp35 (G) HwTx-IV (H) biotin-HTMp7 (I) biotin-HTMp27 (J) biotin-HTMp29

Figure S2. Related to Figure 3. Competition-depended photocrosslinking

(A) FLAG purification stain-free SDS-PAGE. The purified channel was collected in four elutions using the FLAG peptide. (B) Size-exclusion elution profile. Extraction from insect cell membranes and further purification steps required the use of GDN detergent. The fractions were pooled to obtain a homogeneous sample of the tetrameric channel (blue bar). (C) Stain-free SDS-PAGE of VSD2-NavAb after size-exclusion chromatography. The blue bar indicates the fractions that were pooled for photocrosslinking experiments. (D) HTMp29 photocrosslinking. Competition with increasing concentration of HwTx-IV and stain-free SDS-PAGE analysis of molecular weight shift. (E) Biotin-HTMp photoprobes photocrosslinking. Competition with HwTx-IV (150 μM) and stain-free SDS-PAGE analysis of molecular weight shift.

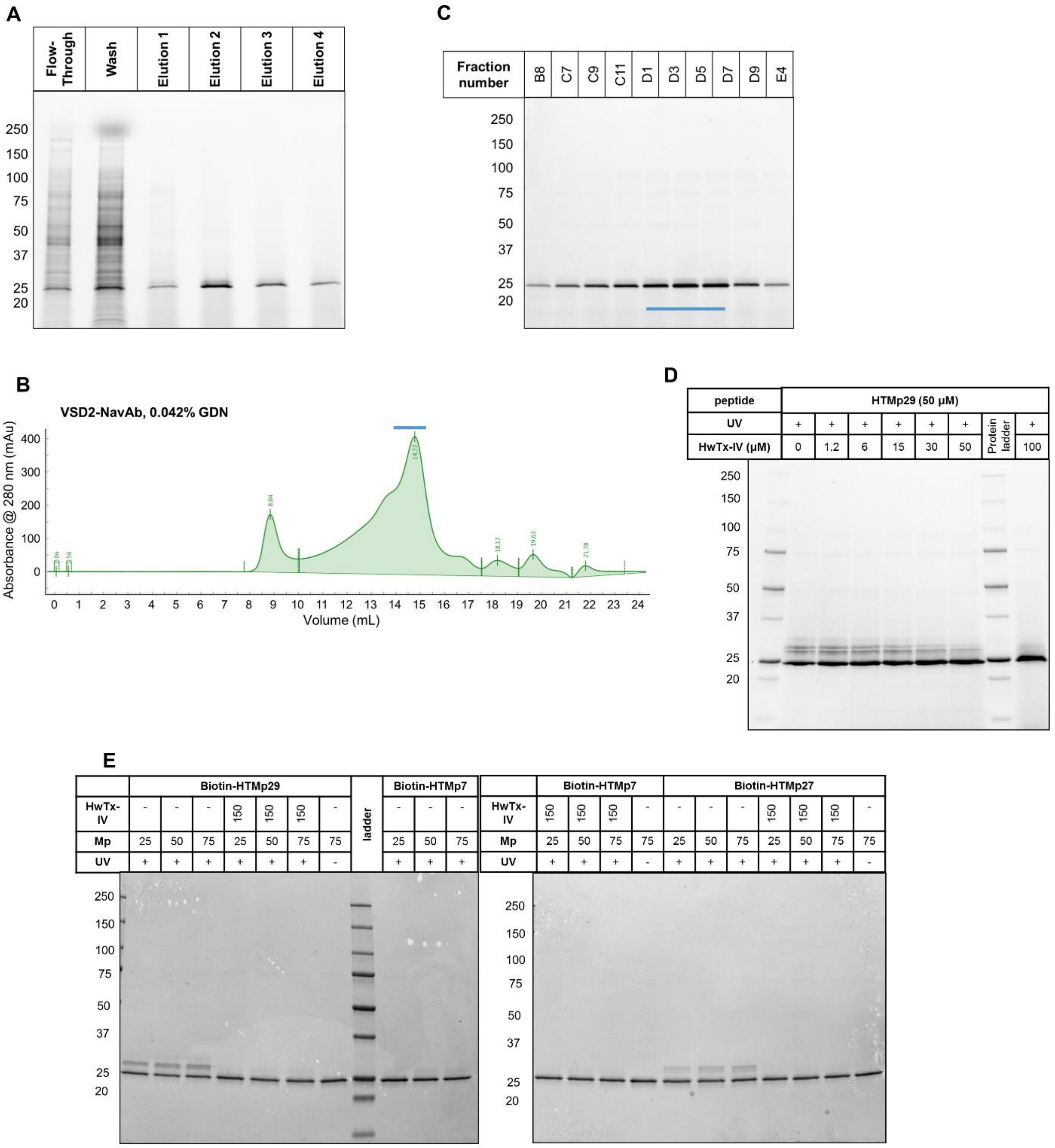

Figure S3. Related to Figure 4. MS spectra of crosslinked peptides

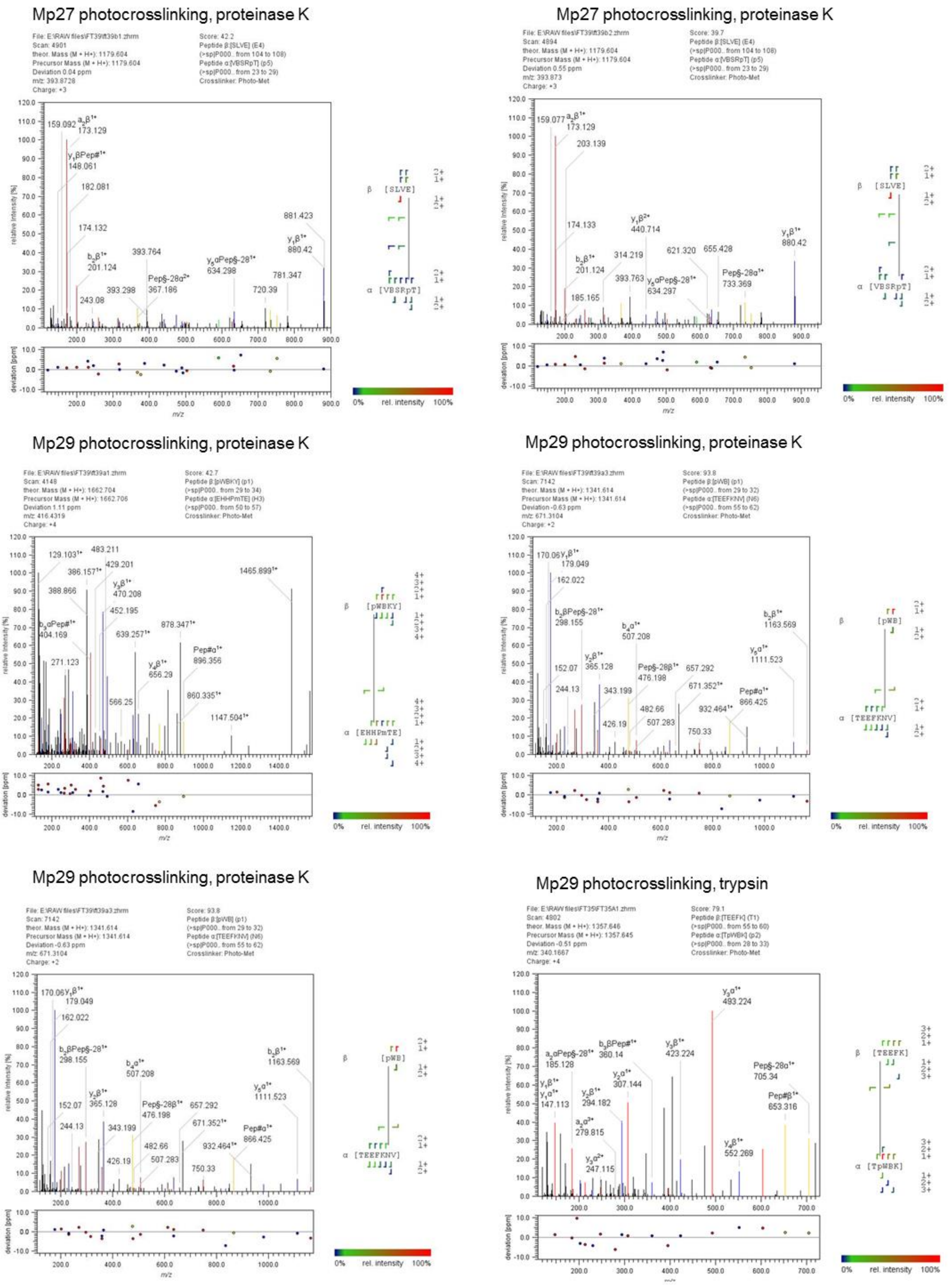

Figure S4. Related to Figure 4. Sequence coverage of VSD2-NavAb with different digestion enzymes

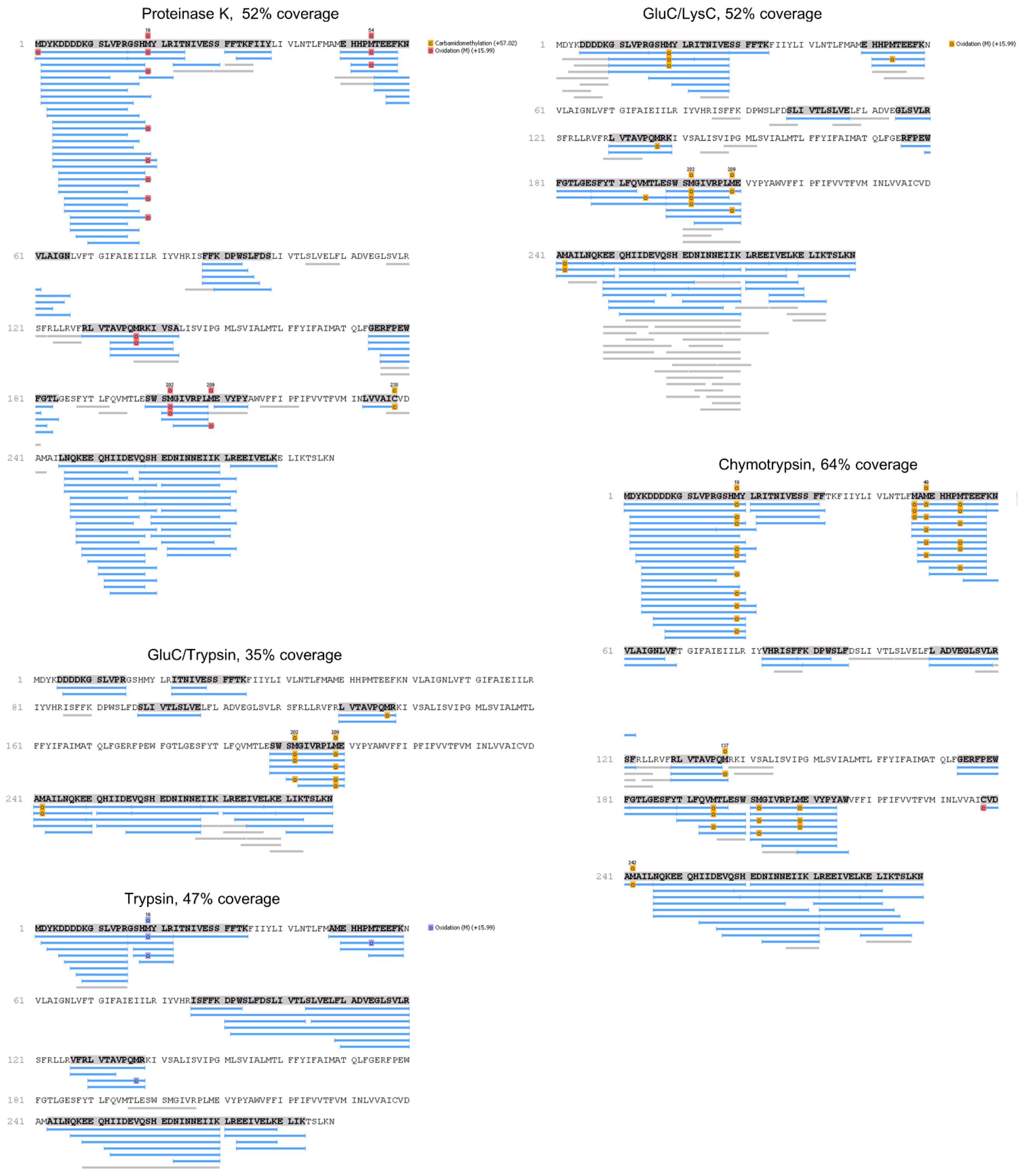

Supplement: Document S1. Figures S1–S4 [file mmc1.pdf]
