# Supplementary material for: Development of Photocrosslinking Probes Based on Huwentoxin-IV to Map the Site of Interaction on Nav1.7
Source: Cell Chem Biol. 2020 Mar 19;27(3):306–313.e4. doi: 10.1016/j.chembiol.2019.10.011 (PMC7083225; doi:10.1016/j.chembiol.2019.10.011)

Brief Communication

Cell Chemical Biology

Development of Photocrosslinking Probes Based on Huwentoxin-IV to Map the Site of Interaction on Nav1.7

Graphical Abstract

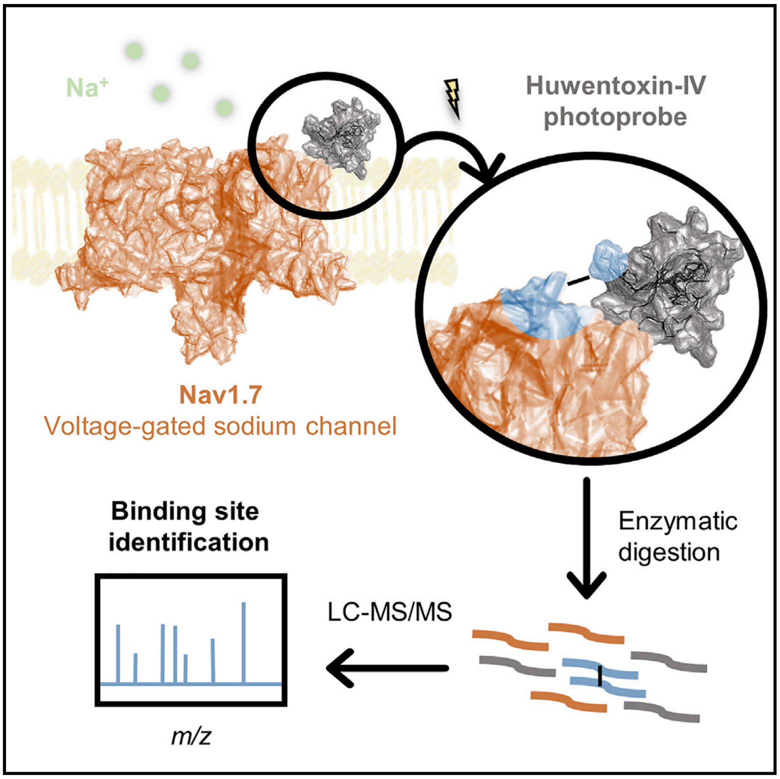

Highlights

- Development of six potent diazirine-containing photoprobes based on Huwentoxin-IV
- Photoprobes specifically photolabel purified bacterial-Nav1.7 VSD2 chimeric channels
- Proteomic mass spectrometry identifies binding site on S1-S2 loop and S3 helix
- Proposed model of HwTx-IV binding reveals importance of K27 and R29

Authors

Foteini Tzakoniati, Hui Xu, Tianbo Li, ..., Jian Payandeh, Christopher M. Koth, Edward W. Tate

Correspondence

e.tate@imperial.ac.uk

In Brief

Classical structural studies of toxin and ion channel complexes are very challenging, and not always feasible. Tzakoniati et al. used photocrosslinking and mass spectrometry to validate and provide direct evidence for specific binding interactions between a toxin peptide and the voltage-gated sodium channel Nav1.7.

Tzakoniati et al., 2020, Cell Chemical Biology *27*, 306–313
March 19, 2020 © 2019 The Authors. Published by Elsevier Ltd.
https://doi.org/10.1016/j.chembiol.2019.10.011

CellPress

Cell Chemical Biology
Brief Communication

Development of Photocrosslinking Probes Based on Huwentoxin-IV to Map the Site of Interaction on Nav1.7

Foteini Tzakoniati,[1] Hui Xu,[2] Tianbo Li,[3] Natalie Garcia,[4] Christine Kugel,[5] Jian Payandeh,[2] Christopher M. Koth,[2] and Edward W. Tate[1,6,*]
[1]Department of Chemistry, Imperial College London, London W12 0BZ, UK
[2]Department of Structural Biology, Genentech, South San Francisco, CA 94080, USA
[3]Department of Biochemical and Cellular Pharmacology, Genentech, South San Francisco, CA 94080, USA
[4]Department of Protein Analytical Chemistry, Genentech, South San Francisco, CA 94080, USA
[5]Department of Biomolecular Resources, Genentech, South San Francisco, CA 94080, USA
[6]Lead Contact
*Correspondence: e.tate@imperial.ac.uk
https://doi.org/10.1016/j.chembiol.2019.10.011

SUMMARY

Voltage-gated sodium (Nav) channels respond to changes in the membrane potential of excitable cells through the concerted action of four voltage-sensor domains (VSDs). Subtype Nav1.7 plays an important role in the propagation of signals in pain-sensing neurons and is a target for the clinical development of novel analgesics. Certain inhibitory cystine knot (ICK) peptides produced by venomous animals potently modulate Nav1.7; however, the molecular mechanisms underlying their selective binding and activity remain elusive. This study reports on the design of a library of photoprobes based on the potent spider toxin Huwentoxin-IV and the determination of the toxin binding interface on VSD2 of Nav1.7 through a photocrosslinking and tandem mass spectrometry approach. Our Huwentoxin-IV probes selectively crosslink to extracellular loop S1-S2 and helix S3 of VSD2 in a chimeric channel system. Our results provide a strategy that will enable mapping of sites of interaction of other ICK peptides on Nav channels.

INTRODUCTION

Voltage-gated sodium (Nav) channels initiate and propagate action potentials in excitable cells by permitting the inward flux of Na^+ ions in response to changes in membrane potential (reviewed in Catterall, 2000). The pore-forming subunit of mammalian Nav channels contain 24-transmembrane segments and are organized as four homologous domains (DI-DIV), each comprising a voltage-sensor domain (VSD) (S1-S4) and two pore-forming helices (S5-S6) (Yan et al., 2017).

Nav1.7 is one of the nine human Nav subtypes, and is expressed in pain-sensing neurons in the peripheral nervous system (Dib-Hajj et al., 2013). Mutations in Nav1.7 are associated with striking pain disorders. For example, gain-of-function mutations cause painful phenotypes such as inherited erythromelalgia and paroxysmal extreme pain disorder (Dib-Hajj et al., 2013), whereas loss-of-function mutations lead to an inability to sense pain, and anosmia, in otherwise healthy individuals (Cox et al., 2006). This genetic evidence highlights the direct involvement of Nav1.7 in pain signaling, and has stimulated intense efforts to discover and develop novel analgesics targeting Nav1.7 that, if sufficiently specific, would presumably not exhibit many of the unfavorable liabilities of current treatments such as those associated with opioids.

Numerous efforts have been undertaken to understand the pharmacological regulation of Nav channels at the molecular level (Clairfeuille et al., 2017). For example, X-ray crystallographic studies have elucidated the binding interactions of aryl sulfonamide inhibitors on Nav1.7, using a chimeric strategy in which the VSD4 of Nav1.7 was fused to the pore of the bacterial *Arcobacter butzleri* Nav channel (NavAb). This strategy enabled high-level production of channel domains suitable for structural studies (Ahuja et al., 2015) and a similar strategy led to the elucidation of the mechanism of VSD2 modulation by inhibitory cystine knot (ICK) peptide Protoxin-II (Xu et al., 2019). High-resolution cryoelectron microscopy (cryo-EM) was employed to map the binding site of the desert bush spider toxin Dc1a onto VSD2 of the American cockroach Nav channel, NavPaS (Shen et al., 2018).

Venoms from spiders are well known for producing modulators of voltage-gated ion channels (Swartz and Mackinnon, 1997). Nav1.7 is potently inhibited by ICK peptides from the tarantula *Ornithoctonus huwena*, such as Huwentoxin-IV (HwTx-IV) (Xiao et al., 2008). Structurally, these peptides adopt an amphipathic architecture, consisting of a hydrophobic and a polar face (Bosmans and Swartz, 2010). Mutagenenesis and patch-clamp electrophysiology experiments suggest that the binding site of HwTx-IV localizes to the extracellular loops of VSD2 on Nav1.7 (Minassian et al., 2013; Xiao et al., 2008), which was more recently observed in the cryo-EM structure of human Nav1.7 in complex with saxitoxin and Huwentoxin-IV (Shen et al., 2019). However, due to the poor resolution of Huwentoxin-IV, the binding interactions with Nav1.7 were not revealed.

An alternative approach involves proteomic mass spectrometry (MS) based identification of crosslinking sites between

 © 2019 The Authors. Published by Elsevier Ltd.
This is an open access article under the CC BY license (http://creativecommons.org/licenses/by/4.0/).

peptide modulators and membrane proteins or ion channels. This approach remains very challenging and has been successful in a limited number of systems, for example, in the ion channel in the nicotinic acetylcholine receptor and α-neurotoxin system (Machold et al., 1995) and, more recently, in a purified neuropeptide Y Y_1 receptor and neuropeptide Y system (Yang et al., 2018). To our knowledge, an analogous study has not been reported to date for a Nav channel binding to an ICK peptide. Parsons and Du Bois synthesized maleimide crosslinkers based on small-molecule Nav pore-blocker saxitoxin; however, identification of the sites of interaction in their subsequent work was indirect, and based on mutagenesis (Parsons and Du Bois, 2013; Thomas-Tran and Du Bois, 2016). Whilst elegant work by Catterall and colleagues determined photocrossinglinking sites for a scorpion toxin using sequence-specific antibodies (Tejedor and Catterall, 1988), the closest comparable example of identification of a photocrosslinking site by mass spectrometry was reported for a simple sevoflurane analog on a voltage-gated potassium channel Kv1.2 (Woll et al., 2017). Further to the exceptional challenges associated with MS-based proteomics of channels and membrane proteins in general and the extremely low levels of recombinant production of human Nav channels, photocrosslinking of peptide binders poses additional challenges with regard to crosslinking site identification due to co-fragmentation in the ligand.

In this study, we have exploited the high affinity of HwTx-IV for the development of chemical tools that probe mechanisms of channel modulation. We present the development of a library of potent photocrosslinking probes of HwTx-IV and demonstrate specific photocrosslinking to a purified Nav1.7 VSD2-NavAb chimeric system by SDS-PAGE shift assays and tandem MS (MS^2). These probes enable direct determination of binding sites of an ICK peptide inhibitor on Nav1.7, provide further insights into the mode of action of HwTx-IV, and reveal new selective binding sites targetable in drug development.

RESULTS AND DISCUSSION

Development of Potent HwTx-IV Toxin Photoprobes Targeting Nav1.7

HwTx-IV is a 35-amino acid, C-terminally amidated ICK peptide with three conserved disulfide bridges (C1-C4, C2-C5, and C3-C6) that stabilize a knotted structure (Figure 1A). The reported half maximal inhibitory concentration (IC_{50}) of HwTx-IV on Nav1.7 is 17 ± 2 nM (Revell et al., 2013) and it displays >2-fold selectivity over other Nav subtypes. A scanning mutagenesis study on HwTx-IV (Revell et al., 2013) identified residues that, when mutated, result in a significant reduction in inhibitory activity, as evaluated by patch-clamp electrophysiology using Chinese hamster ovary (CHO) cells expressing Nav1.7 (Figure 1A). In that work, Revell et al. proposed that residues F6, W30, and Y33, along with the basic residue K32, are important contributors to the affinity and potency of HwTx-IV on Nav1.7 due to the complete loss of antagonist activity upon their substitution to alanine, an observation in agreement with radioligand binding studies (Minassian et al., 2013). Intriguingly, these residues lie on a single face of HwTx-IV (Figure 1B), suggesting that this region directly interacts with Nav1.7.

Taking into account these observations, and the reported NMR structure of HwTx-IV (Peng et al., 2002), we designed HwTx-IV derivatives containing L-photomethionine (Mp) substitutions at positions proximal to F6, W30, K32, and Y33. Mp was chosen because this unnatural amino acid can be readily activated with UV light to form a reactive carbene, and we also hypothesized that the small size of its diazirine head group would not significantly perturb HwTx-IV interactions with Nav1.7. To avoid solely selecting residues that do not participate in binding or are solvent exposed, we chose to investigate positions in close proximity to the presumed binding interface that were previously shown to alter, but not completely eliminate, the inhibitory activity of the peptide in mutagenesis studies (Revell et al., 2013). This "non-essential pharmacophore" consists of I5, K7, K21, K27, R29, and I35 (Figure 1C).

The obtained photoprobes were evaluated in a patch-clamp assay using CHO cells expressing Nav1.7 (Figure 1D). Photoprobes HTMp5, HTMp7, HTMp21, HTMp27, HTMp29, and HTMp35 exhibited IC_{50} values in the range of 13–80 nM. The incorporation of L-photomethionine at these six positions did not significantly affect potency, establishing that the photoprobes behave as reliable mimics of HwTx-IV.

Design and Purification of a Chimeric VSD2-NavAb

For photocrosslinking experiments and to reduce the complexity of working with full-length Nav1.7, we employed a chimera strategy in which hNav1.7 VSD2 sequences were fused to a NavAb scaffold to permit high-level expression and production of stable, purified protein (Ahuja et al., 2015; Xu et al., 2019). This chimeric channel is referred to as VSD2-NavAb and it was used in structural studies of Protoxin-II inhibition (Xu et al., 2019). A similar chimeric channel based on VSD2 and NavAb has exhibited binding to HwTx-IV in surface plasmon resonance assays (Rajamani et al., 2017). The sequence on VSD2-NavAb that corresponds to hNav1.7 VSD2 covers the extracellular regions of S1, S2, S3, and S4 helices, including the S1-S2 and S3-S4 loops, whereas the pore originates from NavAb (Figure 2A). In addition, an amino-terminal FLAG affinity tag was introduced to facilitate purification from insect cells. Following affinity purification, homogeneous NavAb-VSD2 tetrameric channel was resolved by size-exclusion chromatography, yielding a sample of high purity and stability in glyco-diosgenin (GDN) detergent.

Photoprobes HTMp7, HTMp27, and HTMp29 Photocrosslink Efficiently to VSD2-NavAb

Initially, photocrosslinking experiments were performed on purified VSD2-NavAb at a fixed concentration (8.6 μM) and each of the six photoprobes at two concentrations (100 and 300 μM) (Figure 3A), exploring channel to photoprobe molar ratios of 1:5.8 and 1:11.6, respectively. Although photoprobes HTMp5, HTMp21, and HTMp35 did not alter migration of the VSD2-NavAb band by SDS-PAGE analysis, photoactivation in the presence of HTMp7, HTMp27, and HTMp29 led to a shift to higher-molecular weight indicative of photocrosslinking. The apparent molecular weight shift seen for VSD2-NavAb is consistent with covalent modification with photoprobe (~4,000 Da), and western blotting of irradiated and non-irradiated samples using anti-FLAG-HRP revealed that the higher-molecular weight

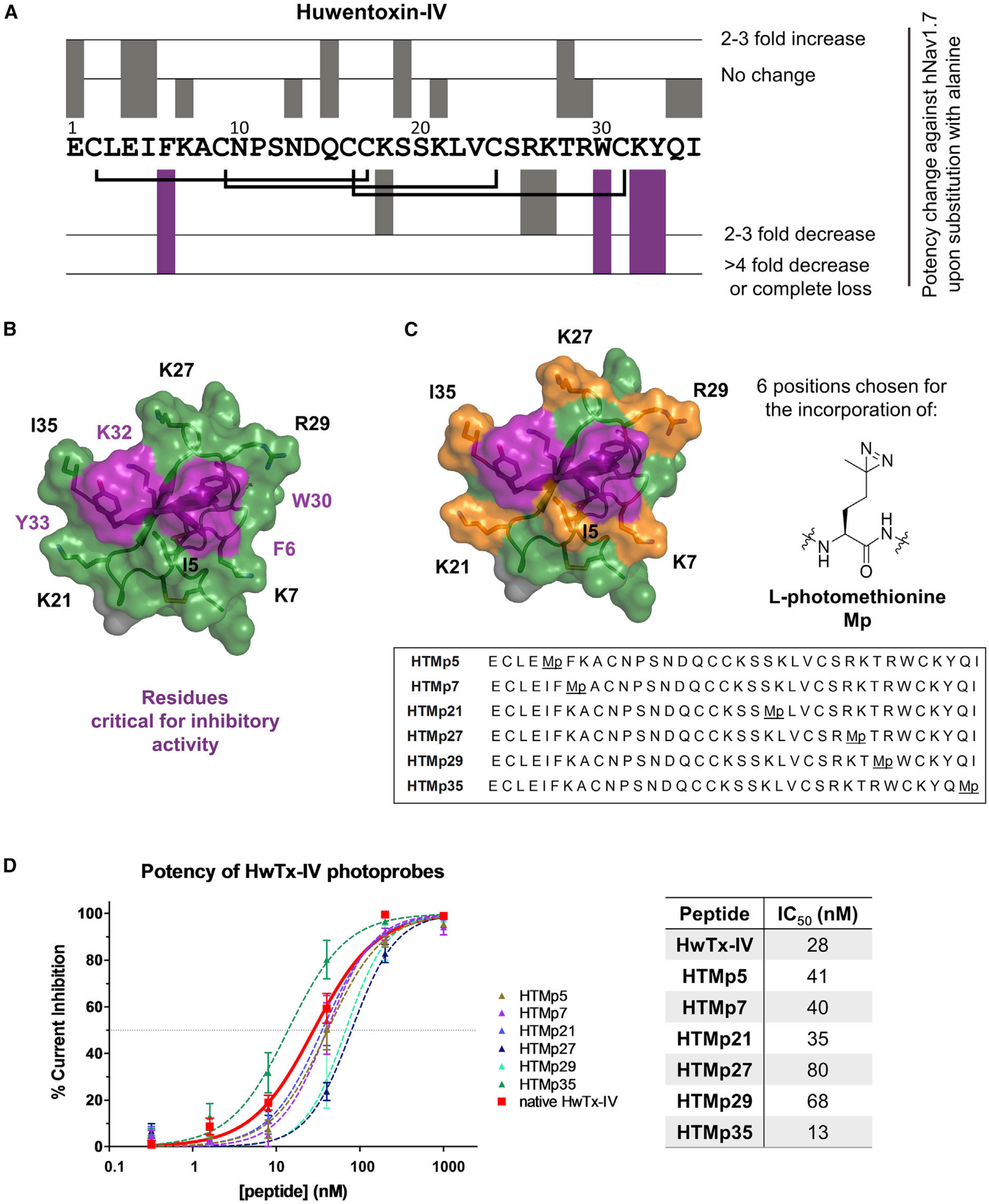

Figure 1. Photoprobe Design and Potency

(A) Sequence of HwTx-IV and alanine scanning mutagenesis information reported by Revell et al. (2013). The bar above or below each residue represents the effect (no change, increase, or decrease) substitution with alanine had to the potency against Nav1.7. Reported native HwTx-IV IC_{50} = 17 ± 2 nM.

(B) Surface representation of HwTx-IV (PDB: 1MB6). The face of interaction with Nav1.7 consists of residues F6, W30, K32, Y33 and is highlighted in purple.

(C) Structure of HwTx-IV (PDB: 1MB6) highlighting the probe design. The residues that were substituted with L-photomethionine in each photoprobe are depicted in orange. The sequences of the photoprobes are listed. See also Figure S1.

(D) Patch-clamp evaluation of HwTx-IV photoprobes. All peptides exhibit IC_{50} values in the range of 13–80 nM with calculated IC_{50} = 28 nM for native HwTx-IV. Data are represented as means ± SEM. See also Figure S1.

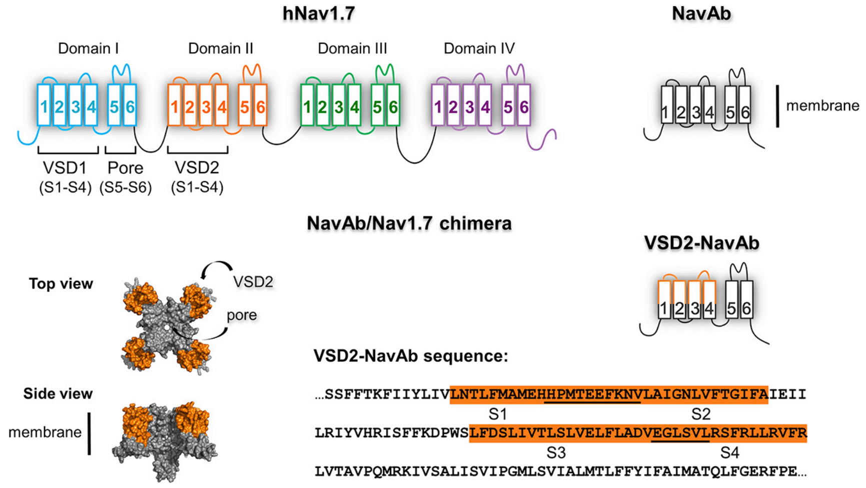

Figure 2. VSD2-NavAb Recombinant Protein Design

Schematic of VSD2-NavAb channel structure and partial amino acid sequence. Each homologous domain of hNav1.7 consists of a voltage sensor (VSD, S1-S4) and pore-forming S5 and S6. Using bacterial NavAb as a scaffold (gray), sequences of hNav1.7 VSD2 were introduced to form a chimeric channel that exhibits the transmembrane and extracellular parts of hNav1.7 VSD2 (in orange). The VSD2-NavAb chimera forms homotetrameric channels with four copies of VSD2 and a NavAb pore. The amino acid sequence of hNav1.7 VSD2 origin in the chimeric channel is highlighted in orange. The extracellular loops are shown in the sequence with a black bar.

band is due to modified VSD2-NavAb (Figure 3B). This effect was achieved at higher concentrations (50 and 100 μM) with respect to the nM potency of these photoprobes, but it is not surprising considering the change in binding context after detergent-facilitated extraction of the VSD2-NavAb from the cell membrane.

Effective photoprobes HTMp27 and HTMp29 exhibited concentration-, irradiation-, and competition-dependent photocrosslinking with substantial efficiency; however, HTMp7 photocrosslinked to VSD2-NavAb less efficiently (Figure 3C). Competition of photocrosslinking in the presence of lower concentrations of the native peptide (HwTx-IV) indicated minimal non-specific photocrosslinking (Figures 3C and S2). It should be noted that the apparent "double" band at the higher molecular weight is not due to multiple photocrosslinking events, but an artifact caused by the specific detergent. A double band at low loading of purified VSD2-NavAb is also observed in SDS-PAGE (Figure S2C). The protein samples are not fully denatured by boiling before loading, therefore it is likely that GDN micelles cause this effect on the visualization of the SDS-PAGE.

Next, N-terminally biotinylated analogs of the three effective photoprobes were synthesized (termed biotin-HTMp7, biotin-HTMp27, and biotin-HTMp29), and their specific crosslinking to VSD2-NavAb evaluated in a streptavidin shift assay (Fairhead and Howarth, 2015; Goya Grocin et al., 2019). For each sample, VSD2-NavAb was treated with 100 μM biotinylated photoprobe, irradiated, and incubated with streptavidin. Complexation with streptavidin results in a substantial molecular weight shift in the SDS-PAGE and western blot of photocrosslinked VSD2-NavAb (Figure 3D), demonstrating that modification is due to photocrosslinking to the photoprobe. In line with previous experiments, efficiency was greatest for probes derived from HTMp27 and HTMp29, with conversion estimated at up to ~50% based on the proportion of protein shifted by streptavidin complexation.

The six positions chosen for incorporation of L-photomethionine cover a broad space around the putative face of interaction (Figure 1B). Although we cannot definitively rule out the effects of detergent on toxin or receptor site stability, the absence of crosslinking for photoprobes HTMp5, HTMp21, and HTMp35 may imply that these positions are not in close proximity to the channel, supported by the observation that positions 7, 27, and 29 (which form crosslinks efficiently when substituted with Mp) are each oriented toward the opposite face of the peptide. The difference in the labeling efficiency for these three photoprobes could also be due to contact with VSD2-NavAb residues that do not react efficiently with the transient carbene upon diazirine photoactivation.

HTMp29 and HTMp27 Crosslink to Specific Regions of S1-S2 and S3

We next sought to identify the sites of modification on VSD2-NavAb to provide insight into the binding mode of the toxin. First, various digestion conditions were evaluated with the aim of achieving high-sequence coverage of unlabeled VSD2-NavAb, focusing particularly on the human Nav1.7 sequence coverage of the chimera (spanning S1-S2 and S3-S4).

The presence of detergent in the protein samples required the use of filter-aided sample preparation (FASP) (Wisniewski et al., 2009), and the resulting peptides were analyzed by nanoscale liquid chromatography-MS^2 (nLC-MS^2) using a Q Exactive spectrometer (Thermo Scientific). Chymotrypsin digestion resulted in the highest sequence coverage (62%), followed by non-specific proteinase K (52%) and GluC/LysC (52%). Although trypsin performed less efficiently, it enabled sufficient detection of the hNav1.7 sequences of interest (Figure S5). Across all digestion enzymes, the achieved sequence coverage was 74%.

The FASP protocol for proteomic sample preparation was employed in the analysis of photocrosslinked VSD2-NavAb with probes HTMp27 (100 μM, 11.6 equivalents.) and HTMp29 (50 μM, 5.8 equivalents.). HTMp29 was introduced at a lower concentration due to the high photocrosslinking efficiency observed by western blotting (Figure 3B). HTMp7 was not selected for this analysis as it displayed low photocrosslinking efficiency in the gel shift assays.

Analysis of irradiated and non-irradiated mixtures of the photoprobe with VSD2-NavAb was performed using MeroX 1.6.6, a software originally designed to identifying crosslinking sites between peptides that result from a MS^2-cleavable crosslinker (Iacobucci et al., 2018), and based on StavroX 3.6.6, which has been used previously in crosslinking studies (Götze et al., 2012). In the case of diazirine photoactivation, the diazo reactive species often reacts with the carboxylic acid in glutamic and

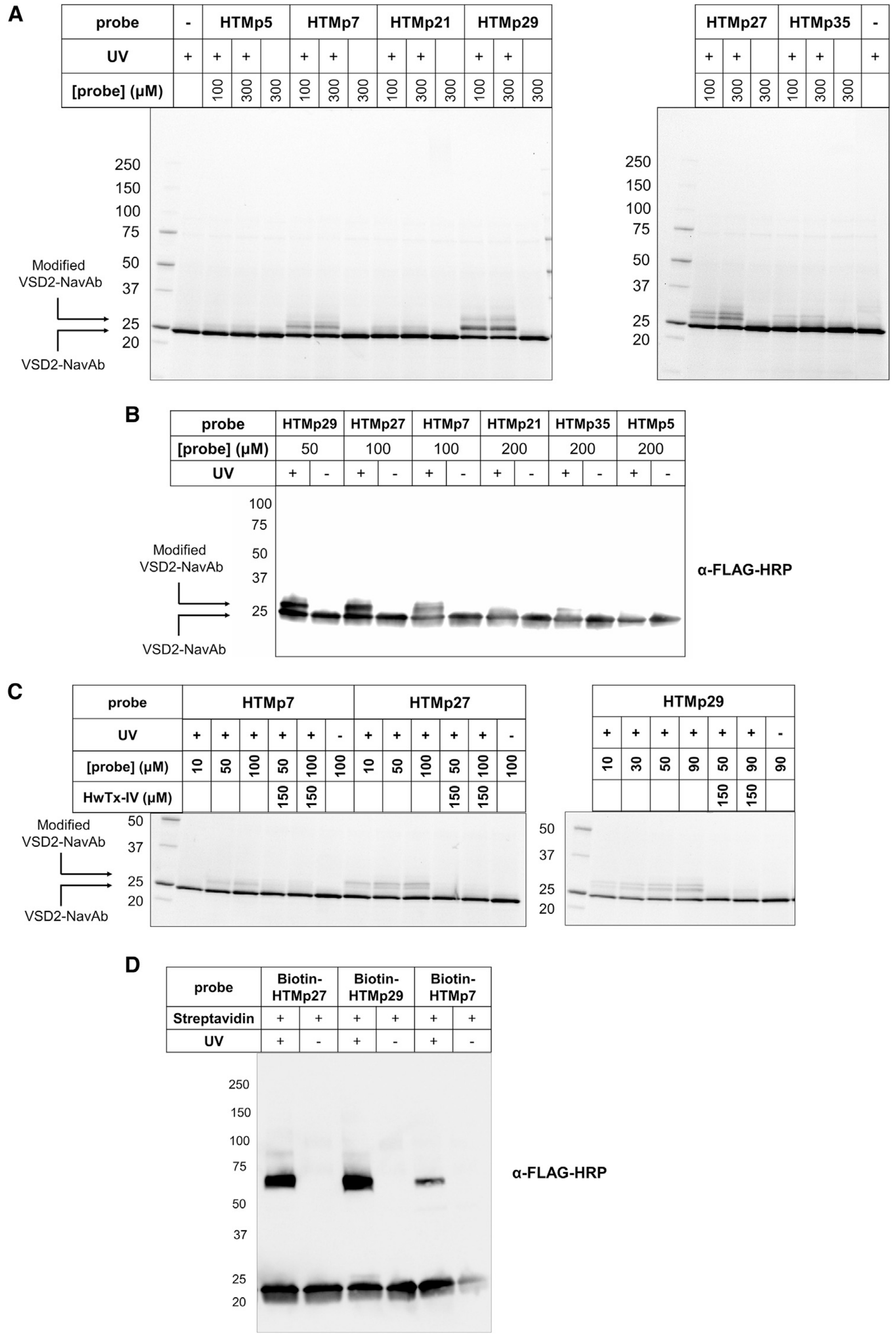

Figure 3. Photocrosslinking Validated by SDS-PAGE Shift Assays

(A) Concentration-dependent photocrosslinking of purified VSD2-NavAb. Photoprobes HTMp7, HTMp27, and HTMp29 demonstrate efficient photocrosslinking by shifting a substantial amount of VSD2-NavAb to higher molecular weight in stain-free SDS-PAGE.

(B) Molecular weight shift by western blot. The molecular weight shift caused by HTMp7, HTMp27, and HTMp29 photocrosslinking is detected by western blotting using anti-FLAG-HRP.

(C) Competition of photocrosslinking with native HwTx-IV. Stain-free SDS-PAGE analysis shows competition by pre-incubation with lower concentrations of HwTx-IV. See also Figure S2.

(D) Use of a streptavidin shift assay to validate photocrosslinking of N terminus biotinylated analogs. Western blotting using anti-FLAG-HRP demonstrates a significant shift in irradiated samples containing streptavidin. HTMp7 photocrosslinking efficiency is significantly lower.

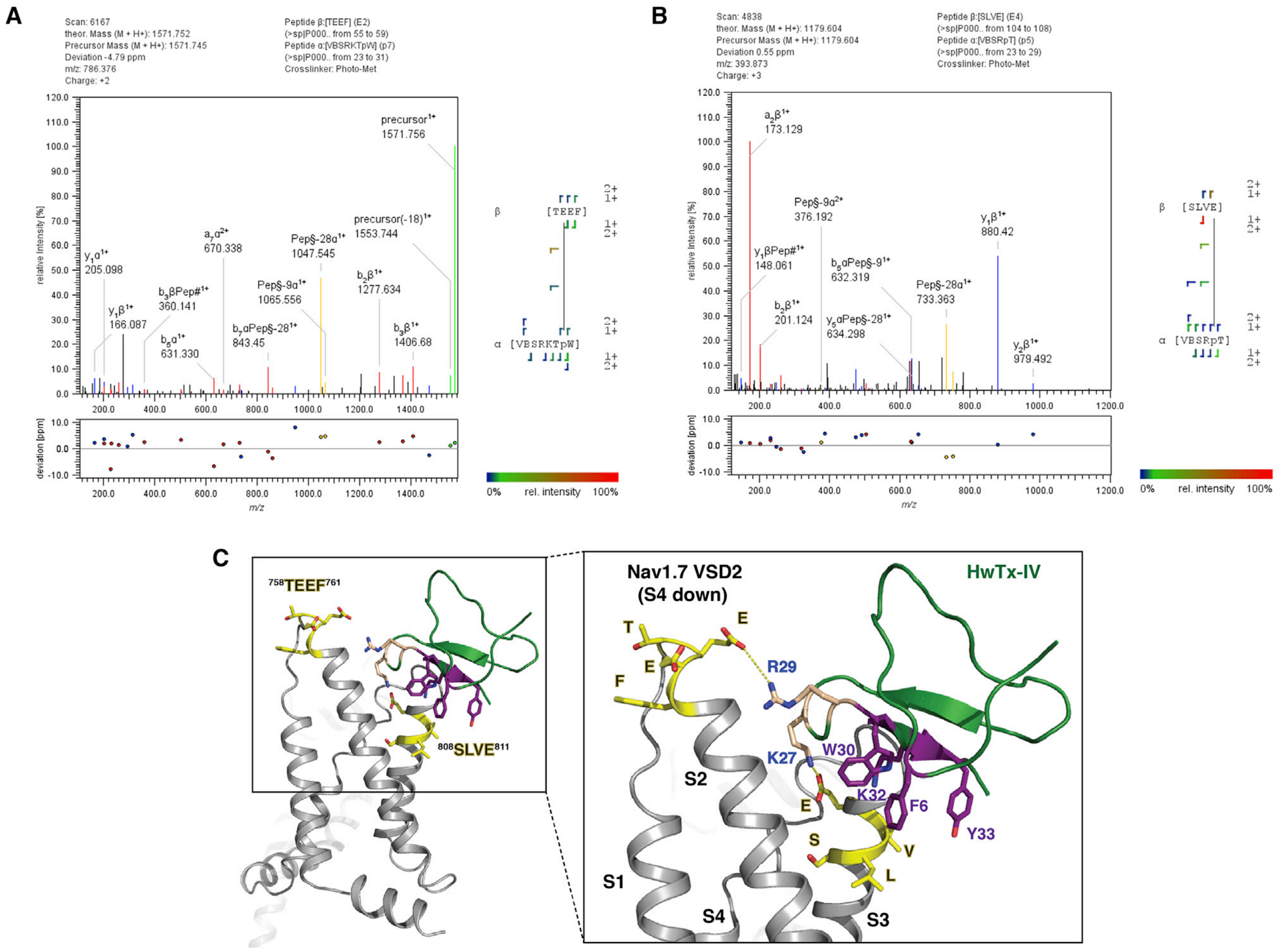

Figure 4. Identification of Sites of Crosslinking

(A) Indicative MS^2 spectrum of HTMp29 crosslinking. The sample was digested using chymotrypsin. MeroX represents L-photomethionine as p found in the sequence of peptide α, and carbamidomethylated cysteine as B. See also Figures S3 and S4.

(B) Indicative MS^2 spectrum of HTMp27 crosslinking. The sample was digested using proteinase K. MeroX represents L-photomethionine as p found in the sequence of peptide α, and carbamidomethylated cysteine as B.

(C) Model of HwTx-IV binding on VSD2-NavAb. The identified crosslinked peptides are highlighted in yellow. The potential site of interaction of residue 29 of HwTx-IV is on the S1-S2 linker loop of VSD2-NavAb. Residue 27 potentially interacts with S3. The VSD2-NavAb structure originates from the cryo-EM studies of VSD2-NavAb in complex with ICK peptide Protoxin-II (Xu et al., 2019).

aspartic acid residues, forming an MS^2-cleavable ester. The resulting *m/z* peak of the initial L-photomethionine-containing peptide α (and the resulting b and y ions) corresponds to a $\Delta m/z$ of -28.0061 (Iacobucci et al., 2018; Pan et al., 2018; Smith et al., 2018). It should be noted that photolysis of a diazirine also yields carbenes that exhibit different reactivity from diazo species (Das, 2011) and do not form MS^2-cleavable products.

For analysis of crosslinking to HTMp27 and HTMp29, trypsin, proteinase K, and chymotrypsin were employed as digestion enzymes. Photoprobe HTMp29 crosslinked consistently to ^{758}TEEF^{761}, ^{758}TEEFK^{762}, ^{753}EHHPMTE^{759}, and ^{758}TEEFKNVL^{765} peptides (depending on the digestion enzyme), which are found in the S1-S2 loop (see Figure 4A for a representative MS^2 spectrum of a chymotrypsin-digested sample and Figure S3 for the remaining spectra). In the case of HTMp27 photocrosslinking, proteinase K digestion resulted in the detection of photocrosslinking to ^{808}SLVE^{811} peptide, part of the S3 helix of VSD2-NavAb (Figures 4B and S3). In all cases, intramolecularly crosslinked peptides were very prominently detected, but this did not hinder detection of intermolecularly crosslinked peptides. This observation is attributed to the excess photoprobe used during incubation and irradiation.

Consistent crosslinking for HTMp29 can be mapped to ^{758}TEEF^{761} on the S1-S2 linker of hNav1.7, supported by multiple peptides from multiple digest conditions, whereas HTMp27 crosslinking was mapped to peptide ^{808}SLVE^{811} in the S3 helix. A proposed model of HwTx-IV interaction with the relevant sequences on VSD2-NavAb (Figure 4C) orients residues F6, W30, K32, and Y33 toward the membrane/transmembrane helices, to enable both S1-S2 and S3 to contact positions 27 and 29. The exact VSD2 residue modified is assigned with moderate confidence by MeroX, with the likelihood of E759 being the site of crosslinking of HTMp29 as 65%, followed by T758 (20%). Indeed, these results are consistent with previous

mutagenesis studies probing the HwTx-IV binding site on Nav1.7, which showed that E753 in the VSD2 S1-S2 loop and E811 in VSD2 S3 were determined to be important residues (Xiao et al., 2011).

In brief, this proposed model suggests that HwTx-IV modulates Nav1.7 in a similar fashion as ProTx-II (Xu et al., 2019); K32 is analogous to K26 of ProTx-II, which interacts with E811 on the S3 helix. This places K27 (analogous to R22 in ProTx-II) in close proximity to ^{808}SLVE^{811}, in contrast to the model that suggests K27 interacts with E818 but not E811 (Minassian et al., 2013). R29 is oriented toward N763 but is sufficiently close to ^{758}TEEF^{761} to allow the carbene to attack the acidic residues upon UV irradiation. Minassian et al. proposed H754 interacts with W30, but our model orients the hydrophobic residues F6, W30, and Y33 to interact with the lipid bilayer. In addition, it suggests R29 as the residue that is close to N774 (N763 in this manuscript) in the cryo-EM structure (Shen et al., 2019). The proposed model illustrates the involvement of the membrane in the HwTx-IV-Nav1.7 complex even though the binding affinity of HwTx-IV to liposomes is weak compared with ProTx-II (Agwa et al., 2017). It further highlights that K27, a residue that is not the most prominent contributor to HwTx-IV inhibitory activity as concluded by alanine scanning mutagenesis studies (Revell et al., 2013), can interact with an acidic residue E811 on S3 helix, while allowing the crucial interaction of K32 with the same residue, and the hydrophobic surface (F6, W30, and Y33) to interact with the lipid bilayer. In addition, HTMp29 photocrosslinking on the S1-S2 loop suggests that HwTx-IV engages Nav1.7 in a fashion which enables a residue at position 29 to interact with the first extracellular loop of VSD2. Future development of Nav1.7 inhibitors with improved potency will benefit from exploring longer side chains at position 29 to facilitate additional interactions with the S1-S2 loop and a variety of basic side chains at position 27 that would further engage S3 helix.

To our knowledge, this is the first report of photocrosslinking site identification between an ICK peptide and a Nav channel. This study demonstrates the development and successful application of potent site-specific photoprobes based on peptide modulators of a membrane protein. Important steps in this approach involve the use of a highly purified recombinant channel that remains stable in detergent during photocrosslinking, the exploration of various positions for the introduction of L-photomethionine in the toxin, the optimization of digestion and sample preparation conditions, along with MeroX analysis for the identification of crosslinking sites. In the pursuit of binding sites on sensory ion channels, our study further demonstrates that photocrosslinking coupled with nLC-MS^2 can provide an orthogonal technique for interpretation of structural and mutagenesis data.

SIGNIFICANCE

Identification of toxin binding sites on the voltage sensor domain of voltage-gated sodium channels remains a significant challenge in structural biology and electrophysiology studies. Focusing on the pain-associated hNav1.7 channel, we developed a new library of six diazirine-photoprobes based on the ICK peptide Huwentoxin-IV. Efficient photocrosslinking to a purified chimeric hNav1.7 VSD2-NavAb channel was exhibited by photoprobes HTMp7, HTMp27, and HTMp29, and this was further validated by competition assays and the ability of biotinylated analogs to significantly alter the migration of VSD2-NavAb in streptavidin shift assays. Subsequently, we identified the crosslinking sites of HTMp27 and HTMp29 on the S3 helix and S1-S2 linker of the voltage sensor, respectively, enabling us to propose a new model for HwTx-IV binding based on the sites of labeling and a homology model of full-length Nav1.7 channel. This work paves the way for the development and application of photoprobes based on other gating modifier toxins to understand the mechanisms behind channel modulation through interaction with voltage sensor domains.

STAR★METHODS

Detailed methods are provided in the online version of this paper and include the following:

- KEY RESOURCES TABLE
- LEAD CONTACT AND MATERIALS AVAILABILITY
- METHOD DETAILS
  - Peptide Synthesis and Purification
- EXPERIMENTAL MODEL AND SUBJECT DETAILS
  - Patch-Clamp Electrophysiology
  - Photocrosslinking
  - SDS-PAGE and Western Blotting
  - Streptavidin Shift Assay
  - Proteomic Sample Preparation
  - nLC-MS/MS
  - PEAKS Data Analysis
  - MeroX Data Analysis
- QUANTIFICATION AND STATISTICAL ANALYSIS
- DATA AND CODE AVAILABILITY

SUPPLEMENTAL INFORMATION

Supplemental Information can be found online at https://doi.org/10.1016/j.chembiol.2019.10.011.

ACKNOWLEDGMENTS

We would like to thank Dr. Chiara Fabbro and Dr. Remigiusz Serwa for collecting MS data. We thank Smartox Biotechnology for generation of HwTx-IV reagents used in this study. F.T. gratefully acknowledges support from the Medical Research Council (Proximity to Discovery Award, MC_PC_14128) and the Biotechnology and Biosciences Research Council (BBSRC) (grant BB/L50239X/1).

AUTHOR CONTRIBUTIONS

F.T., C.M.K., and E.W.T. designed the research. F.T. and T.L. performed the research. F.T. analyzed the data. H.X. developed the model of binding. F.T., C.M.K., and E.W.T. wrote the manuscript. H.X. and C.K. contributed DNA reagents and performed protein expression. H.X., N.G., J.P., C.M.K., and E.W.T. provided expertise and feedback. E.W.T. directed the research.

DECLARATION OF INTERESTS

F.T., H.X., T.L., N.G., C.K., J.P., and C.M.K. are/were Genentech employees. T.L., J.P., and C.M.K. hold shares in the Genentech/Roche group. E.W.T. is a Director and holds shares in Myricx Pharma Ltd.

Received: February 22, 2019
Revised: August 31, 2019
Accepted: October 24, 2019
Published: November 12, 2019

REFERENCES

Agwa, A.J., Lawrence, N., Deplazes, E., Cheneval, O., Chen, R., Craik, D.J., Schroeder, C.I., and Henriques, S.T. (2017). Spider peptide toxin HwTx-IV engineered to bind to lipid membranes has an increased inhibitory potency at human voltage-gated sodium channel hNaV1.7. Biochim. Biophys. Acta Biomembr. *1859*, 835–844.

Ahuja, S., Mukund, S., Deng, L., Khakh, K., Chang, E., Ho, H., Shriver, S., Young, C., Lin, S., Johnson, J.P., Jr., et al. (2015). Structural basis of Nav1.7 inhibition by an isoform-selective small-molecule antagonist. Science *350*, aac5464.

Bosmans, F., and Swartz, K.J. (2010). Targeting voltage sensors in sodium channels with spider toxins. Trends Pharmacol. Sci. *31*, 175–182.

Catterall, W.A. (2000). From ionic currents to molecular mechanisms: the structure and function of voltage-gated sodium channels. Neuron *26*, 13–25.

Clairfeuille, T., Xu, H., Koth, C.M., and Payandeh, J. (2017). Voltage-gated sodium channels viewed through a structural biology lens. Curr. Opin. Struct. Biol. *45*, 74–84.

Cox, J.J., Reimann, F., Nicholas, A.K., Thornton, G., Roberts, E., Springell, K., Karbani, G., Jafri, H., Mannan, J., Raashid, Y., et al. (2006). An SCN9A channelopathy causes congenital inability to experience pain. Nature *444*, 894–898.

Das, J. (2011). Aliphatic diazirines as photoaffinity probes for proteins: recent developments. Chem. Rev. *111*, 4405–4417.

Dib-Hajj, S.D., Yang, Y., Black, J.A., and Waxman, S.G. (2013). The NaV1.7 sodium channel: from molecule to man. Nat. Rev. Neurosci. *14*, 49–62.

Fairhead, M., and Howarth, M. (2015). Site-specific biotinylation of purified proteins using BirA. Methods Mol. Biol. *1266*, 171–184.

Götze, M., Pettelkau, J., Schaks, S., Bosse, K., Ihling, C.H., Krauth, F., Fritzsche, R., Kühn, U., and Sinz, A. (2012). StavroX-A software for analyzing crosslinked products in protein interaction studies. J. Am. Soc. Mass Spectrom. *23*, 76–87.

Goya Grocin, A., Serwa, R.A., Morales Sanfrutos, J., Ritzefeld, M., and Tate, E.W. (2019). Whole proteome profiling of N-myristoyltransferase activity and inhibition using Sortase-A. Mol. Cell. Proteomics *18*, 115–126.

Iacobucci, C., Goetze, M., Piotrowski, C., Arlt, C., Rehkamp, A., Ihling, C.H., Hage, C., and Sinz, A. (2018). Carboxyl-photo-reactive MS-cleavable cross-linkers: unveiling a hidden aspect of diazirine-based reagents. Anal. Chem. *90*, 2805–2809.

Machold, J., Utkin, Y., Kirsch, D., Kaufmann, R., Tsetlin, V., and Hucho, F. (1995). Photolabeling reveals the proximity of the alpha-neurotoxin binding site to the M2 helix of the ion channel in the nicotinic acetylcholine receptor. Proc. Natl. Acad. Sci. U S A *92*, 7282–7286.

Minassian, N.A., Gibbs, A., Shih, A.Y., Liu, Y., Neff, R.A., Sutton, S.W., Mirzadegan, T., Connor, J., Fellows, R., Husovsky, M., et al. (2013). Analysis of the structural and molecular basis of voltage-sensitive sodium channel inhibition by the spider toxin huwentoxin-IV (mu-TRTX-Hh2a). J. Biol. Chem. *288*, 22707–22720.

Pan, D., Brockmeyer, A., Mueller, F., Musacchio, A., and Bange, T. (2018). A simplified protocol for cross-linking mass spectrometry using the MS-cleavable cross-linker DSBU with efficient cross-link identification. Anal. Chem. *90*, 10990–10999.

Parsons, W.H., and Du Bois, J. (2013). Maleimide conjugates of saxitoxin as covalent inhibitors of voltage-gated sodium channels. J. Am. Chem. Soc. *135*, 10582–10585.

Peng, K., Shu, Q., Liu, Z., and Liang, S. (2002). Function and solution structure of huwentoxin-IV, a potent neuronal tetrodotoxin (TTX)-sensitive sodium channel antagonist from Chinese bird spider *Selenocosmia huwena*. J. Biol. Chem. *277*, 47564–47571.

Rajamani, R., Wu, S., Rodrigo, I., Gao, M., Low, S., Megson, L., Wensel, D., Pieschl, R., Post-Munson, D., Watson, J., et al. (2017). A functional NaV1.7-NavAb chimera with a reconstituted high affinity ProTx-II binding site. Mol. Pharmacol. *92*, 310–317.

Revell, J.D., Lund, P.E., Linley, J.E., Metcalfe, J., Burmeister, N., Sridharan, S., Jones, C., Jermutus, L., and Bednarek, M.A. (2013). Potency optimization of Huwentoxin-IV on hNav1.7: a neurotoxin TTX-S sodium-channel antagonist from the venom of the Chinese bird-eating spider *Selenocosmia huwena*. Peptides *44*, 40–46.

Shen, H., Li, Z., Jiang, Y., Pan, X., Wu, J., Cristofori-Armstrong, B., Smith, J.J., Chin, Y.K.Y., Lei, J., Zhou, Q., et al. (2018). Structural basis for the modulation of voltage-gated sodium channels by animal toxins. Science *2596*, 16–18.

Shen, H., Shen, H., Liu, D., Wu, K., Lei, J., and Yan, N. (2019). Structures of human Nav1.7 channel in complex with auxiliary subunits and animal toxins. Science *363*, 1303–1308.

Smith, D.L., Götze, M., Bartolec, T.K., Hart-Smith, G., and Wilkins, M.R. (2018). Characterization of the interaction between arginine methyltransferase Hmt1 and its substrate Npl3: use of multiple cross-linkers, mass spectrometric approaches, and software platforms. Anal. Chem. *90*, 9101–9108.

Swartz, K.J., and Mackinnon, R. (1997). Mapping the receptor site for hanatoxin, a gated modifier of voltage-dependent K+ cannels. Neuron *18*, 675–682.

Tejedor, F.J., and Catterall, W.A. (1988). Site of covalent attachment of alpha-scorpion toxin derivatives in domain I of the sodium channel alpha subunit. Proc. Natl. Acad. Sci. U S A *85*, 8742–8746.

Thomas-Tran, R., and Du Bois, J. (2016). Mutant cycle analysis with modified saxitoxins reveals specific interactions critical to attaining high-affinity inhibition of hNav1.7. Proc. Natl. Acad. Sci. U S A *113*, 5856–5861.

Wisniewski, J.R., Zougman, A., Nagaraj, N., and Mann, M. (2009). Universal sample preparation method for proteome analysis. Nat. Methods *6*, 359–362.

Woll, K.A., Peng, W., Liang, Q., Zhi, L., Jacobs, J.A., Maciunas, L., Bhanu, N., Garcia, B.A., Covarrubias, M., Loll, P.J., et al. (2017). Photoaffinity ligand for the inhalational anesthetic sevoflurane allows mechanistic insight into potassium channel modulation. ACS Chem. Biol. *12*, 1353–1362.

Wollscheid, B., Bausch-Fluck, D., Henderson, C., O'Brien, R., Bibel, M., Schiess, R., Aebersold, R., and Watts, J.D. (2009). Mass-spectrometric identification and relative quantification of N-linked cell surface glycoproteins. Nat. Biotechnol. *27*, 378–386.

Xiao, Y., Bingham, J.-P., Zhu, W., Moczydlowski, E., Liang, S., and Cummins, T.R. (2008). Tarantula huwentoxin-IV inhibits neuronal sodium channels by binding to receptor site 4 and trapping the domain II voltage sensor in the closed configuration. J. Biol. Chem. *283*, 27300–27313.

Xiao, Y., Jackson, J.O., Liang, S., and Cummins, T.R. (2011). Common molecular determinants of tarantula huwentoxin-IV inhibition of Na^+ channel voltage sensors in domains II and IV. J. Biol. Chem. *286*, 27301–27310.

Xu, H., Li, T., Rohou, A., Arthur, C.P., Tzakoniati, F., Wong, E., Estevez, A., Kugel, C., Franke, Y., Chen, J., et al. (2019). Structural basis of Nav1.7 inhibition by a gating-modifier spider toxin. Cell *176*, 702–715.

Yan, Z., Zhou, Q., Wang, L., Wu, J., Zhao, Y., Huang, G., Peng, W., Shen, H., Lei, J., and Yan, N. (2017). Structure of the Nav1.4-β1 complex from electric eel. Cell *170*, 470–482.

Yang, Z., Han, S., Keller, M., Kaiser, A., Bender, B.J., Bosse, M., Burkert, K., Kögler, L.M., Wifling, D., Bernhardt, G., et al. (2018). Structural basis of ligand binding modes at the neuropeptide Y Y1 receptor. Nature *556*, 520–524.

STAR★METHODS

KEY RESOURCES TABLE

REAGENT or RESOURCE	SOURCE	IDENTIFIER
Antibodies		
mouse monoclonal anti-FLAG M2-HRP	Sigma	Cat# A8592; RRID: AB_439702
Biological Samples		
Tni cells	Expression Systems	Cat# 94-002F
CHO Nav1.7 cells	Anaxon	N/A
Recombinant DNA		
VSD2-NavAb	This study	DNA766564
Chemicals, Peptides, and Recombinant Proteins		
EDTA-free complete protease inhibitors	Roche	Cat# 11873580001
GDN	Anatrace	Cat# GDN101
anti-FLAG M2 agarose resin	Sigma	Cat# A2220
FLAG peptide	Sigma	Cat# F3290
streptavidin	Jackson Immunoresearch	Cat# 016-000-084
1,4-Dithiothreitol	Sigma	Cat# 43815
Crescendo Western HRP substrate	EMD Millipore	Cat# WBLUR0100
trypsin	Promega	Cat# V5111
Glu-C	Promega	Cat# V1651
proteinase K	New England Biolabs	Cat# P8107
chymotrypsin	Promega	Cat# V106A
ProteaseMAX	Promega	Cat# V2071
HTMp peptides and biotinylated analogues	Smartox Biotech	N/A
Deposited Data		
VSD2-ProTx2 structure	Xu et al., 2019	PDB: 6N4R
Raw and analysed nLC-MS/MS data	This study, and http://proteomecentral.proteomexchange.org	Dataset identifier PXD015037
Huwentoxin-IV structure	Peng et al., 2002	PDB: 1MB6
Software and Algorithms		
PEAKS 8.5	Bionformatics Solutions	http://www.bioinfor.com/peaks8-5/
GraphPad Prism v6.05	GraphPad	https://www.graphpad.com/scientific-software/prism/
PyMOL	Schrödinger, LLC	https://pymol.org/2/
MeroX 1.6.6	Iacobucci et al., 2018	https://www.stavrox.com/
Other		
100 KDa MWCO Amicon-15 concentrator	Merck	Cat# 10447871
Superose 6 Increase 10/300 GL column	GE Healthcare	Cat# 29091596
Nunc 96 well plate	Thermo Scientific	Cat# 167311
Spectrolinker XL1500	Spectronics Corporation	N/A
4–20% Stain Free Mini-PROTEAN TGX	Bio-Rad	Cat# 4568095
Gel Doc EZ System	Bio-Rad	N/A
30 kDa MWCO Vivacon 500 concentrator	Sartorius	Cat# 518-0002
SDB-XC poly(styrenedivinylbenzene) copolymer	3M	Cat# 2240
Nitrocellulose membrane	GE Healthcare	Cat# 10600003

LEAD CONTACT AND MATERIALS AVAILABILITY

Further information and requests for reagents should be directed to and will be fulfilled by the Lead Contact, Edward W. Tate (e.tate@imperial.ac.uk). The photoprobes and Tni cells are available from the Lead Contact without restriction. This study did not generate other new unique reagents.

METHOD DETAILS

Peptide Synthesis and Purification

The photoprobes were obtained commercially from Smartox Biotechnology, France. All linear peptides were assembled stepwise by standard Fmoc SPPS chemistry on a Symphony Synthesizer (Protein technologies Inc.) using a 2-chlorotrityl chloride resin substituted with Ram linker, substitution rate of 1.6 mmol g^{-1}. After resin cleavage and deprotection in trifluoroacetic acid/water/triisopropyl silane/1,3-dimethoxybenzene (92.5:2.5:2.5:2.5 % v/v), crude toxin analogues were folded by air oxidation in Tris buffer in presence of the reduced glutathione (GSH) and oxidized glutathione (GSSG) redox couple. The resulting oxidized peptides were purified to homogeneity by C18 reversed phase chromatography (RP-HPLC) on a Jupiter Proteo column (Phenomenex, 4 μm, 21.2 mm ID x 250 mm L) using an Agilent Technologies preparative HPLC system (1260 Infinity) and characterized by accurate mass quadrupole time-of-flight mass spectrometry with electrospray ionization (LC-ESI Q-TOF-MS) (Waters Q-TOF Xevo G2S mass spectrometer equipped with an Acquity UHPLC system).

EXPERIMENTAL MODEL AND SUBJECT DETAILS

For electrophysiological recordings, the human Nav1.7 (XM_011511618) expressing CHO stable cell line (Anaxon, Switzerland) was cultured in Ham's F12 medium supplemented with 10% FBS, 2 mM L-Glutamine and required selection antibiotic in 5% CO_2 at 37°C.

For VSD2-NavAb chimera expression and purification, recombinant baculovirus was generated using the Baculogold system (BD Biosciences) following standard protocols. *Trichoplusia ni* (Tni) cells were infected for protein production and harvested 48 h post-infection. The sequence of VSD2-NavAb is given below. The FLAG affinity tag and thrombin protease cleavage site are underlined. The sequence originating from hNav1.7 VSD2 is highlighted in bold.

<u>MDYKDDDDKGSLVPRGSH</u>MYLRITNIVESSFFTKFIIYLIV**LNTLFMAMEHHPMTEEFKNVLAIGNLVFTGIFA**IEIILRIYVHRISFFKDPWS**LFDSLIVTLSLVELFLADVEGLSVLRSFRLLRVFR**LVTAVPQMRKIVSALISVIPGMLSVIALMTLFFYIFAIMATQLFGERFPEWFGTLGESFYTLFQVMTLESWSMGIVRPLMEVYPYAWVFFIPFIFVVTFVMINLVVAICVDAMAILNQKEEQHIIDEVQSHEDNINNEIIKLREEIVELKELIKTSLKN

Patch-Clamp Electrophysiology

For all Nav channel recordings, the intracellular solution contained (in mM): 50 CsCl, 60 CsF, 10 NaCl, 20 EGTA and 10 HEPES (pH 7.2, osmolarity 285 mOsm), and extracellular solution contained (in mM): 80 NaCl, 60 NMDG, 4 KCl, 2 $CaCl_2$, 1 $MgCl_2$, 5 glucose and 10 HEPES (pH 7.4, osmolarity 300 mOsm). Automated patch clamp (APC) recordings were performed by using SyncroPatch 768PE (Nanion Technologies, Germany), with PatchController384 V1.5.2 and DataController384 V1.5.0 software for pulse generation and data collection. Currents were sampled at 10 kHz and filtered with Bessel filter. Series resistance was compensated 80% with leak subtraction. Seal resistance (Rseal) was calculated using built-in protocols, and high-quality recoding data was obtained by auto filtering 3 quality control criteria: cell catching (>10 MΩ), seal resistance (>500 MΩ) and baseline current amplitude (>500 pA), and follow with manual inspection. The voltage protocol for all toxin pharmacological tests was set with holding membrane potential (Vm) at -120 mV, eliciting a 10 ms pulse to -10 mV every 2 s, recording 5 min for baseline and followed with examining compound effect for 25 min. Nav channel inhibition was calculated as the percentage of peak current (I) decrease from before compound application (I_Baseline) to the end of 25 minutes compound application (I_End) and both being normalized to the end of experiment full block current (I_Fullblock) using this following equation:

$$Block\% = \left[1 - \left(\frac{I_{end} - I_{fullblock}}{I_{baseline} - I_{fullblock}}\right)\right] \times 100\%$$

Photocrosslinking

The purified VSD2-NavAb chimera (0.3 mg mL^{-1}, 8.6 μM in 10 mM Tris pH 8.0 and 100 mM NaCl and 0.042% (w/v) GDN) was incubated with the photoprobes: Mp5 (200 μM), Mp7 (100 μM), Mp21 (200 μM), Mp27 (100 μM), Mp29 (50 μM) and Mp35 (200 μM) on ice for 1 h. The samples were transferred to a transparent 96 well plate (Nunc, Thermo Scientific) and irradiated at 365 nm in a UV box (Spectrolinker XL1500, Spectronics Corporation) for 10 min on ice. The competition with native Huwentoxin-IV was done with pre-incubation for 30 min on ice.

SDS-PAGE and Western Blotting

1-5 μg of protein samples were incubated with 1x Laemmli SLB supplemented with 10% v/v v/v β-mercaptoethanol and loaded into 4–20% polyacrylamide Mini-PROTEAN TGX gels (200 mV for 45 min). The gels were visualised using a Bio-Rad Gel Doc EZ System.

Gels were briefly washed with deionised water and the proteins were transferred to a 0.45 μm Nitrocellulose membrane (GE Healthcare) in transfer buffer containing 25 mM Tris, 190 mM glycine and 20% v/v methanol. TBST buffer (20 mM Tris pH 7.5, 150 mM NaCl, 0.1% Tween-20) was used for all washes and resuspension of blocking reagents and antibodies. Membranes were blocked at RT for 1 h with skim milk powder (5 % w/v in TBST). Incubation with anti-FLAG M2-HRP mouse monoclonal (Sigma) was done for 1 h in blocking solution and then the membrane was washed with TBST (3 x 10 min). Detection was carried out using Crescendo Western HRP substrate (Millipore) according to the manufacturer's instructions and on an ImageQuant LAS4000.

Streptavidin Shift Assay

After UV irradiation, protein samples (5 μL) were incubated with 4 μL streptavidin (Jackson Immunoresearch, 100 μM in water) in 1 x Laemmli sample loading buffer supplemented with 10% v/v β-mercaptoethanol at RT for 10 min. The samples were then loaded on an SDS-PAGE gel (4–20% Stain Free Mini-PROTEAN TGX™, Bio-Rad) and electrophoresis was performed in a Tris/Glycine/SDS system at 200 mV for 45 min. The gels were visualised using a Bio-Rad Gel Doc EZ System.

Proteomic Sample Preparation

The FASP protocol (Wisniewski et al., 2009) was employed with some modifications to prepare proteomics samples from purified and photocrosslinked VSD2-NavAb. More specifically, the samples were reduced with dithiothreitol (10 mM) for 30 min at 55°C and then transferred to a 30 kDa MWCO Vivacon 500 concentrator (Sartorius). Urea solution (200 μL, 8 M urea in 100 mM Tris pH 8.5) was added and the samples were centrifuged at 14000*g* for 10 min. Iodoacetamide was added to a final concentration of 50 mM and the mixture was vortexed for 1 min before incubation in the dark for 20 min. The concentrators were washed three times with urea solution and three times with AMBIC solution (100 μL, 50 mM NH4HCO3 pH 8.5). Digestion was performed using sequencing grade modified trypsin (Promega), Glu-C (Promega), Proteinase K (New England Biolabs) or chymotrypsin (Promega) with either a 1:50 or 1:100 w/w enzyme/protein ratio and 0.05% w/v ProteaseMAX (Promega). All samples were shaken overnight at 37°C except for proteinase K (37°C, 3 h) and chymotrypsin (25°C, overnight). Digestion peptides were collected into a clean Vivacon tube with 40 μL washes of AMBIC solution (50 mM) and NaCl (0.5 M). The peptides were acidified with 0.5% v/v trifluoroacetic acid (TFA) and desalted using StageTip method (Wollscheid et al., 2009). Elution from the sorbent (SDB-XC poly(styrenedivinylbenzene) copolymer, from 3M) with 79% v/v MeCN in water was followed by speed-vac assisted solvent removal.

nLC-MS/MS

The analysis was performed using an Acclaim PepMap RSLC column 50 cm x 75 mm inner diameter (Thermo Fisher Scientific) using a 1 h MeCN gradient (2–55% v/v) in 0.1% v/v aqueous formic acid at a flow rate of 250 nL min^{-1}. Easy nLC-1000 was coupled to a Q Exactive mass spectrometer via an easy-spray source (all Thermo Fisher Scientific). The Q Exactive was operated in data-dependent mode with survey scans acquired at a resolution of 75,000 at *m/z* 200 (transient time 256 ms). Up to 10 of the most abundant isotope patterns with charge 2+ or higher from the survey scan were selected with an isolation window of 3.0 *m/z* and fragmented by higher energy collision dissociation (HCD) with normalized collision energies of 25. The maximum ion injection times for the survey scan and the MS/MS scans (acquired with a resolution of 17,500 at *m/z* 200) were 20 and 120 ms, respectively. The ion target value for MS was set to 10^6 and for MS/MS to 10^5, and the intensity threshold was set to 8.3×10^2.

PEAKS Data Analysis

PEAKS 8.5 was used for the analysis of VSD2-NavAb. The database search was performed against the sequence of VSD2-NavAb. Cysteine carbamidomethylation was selected as a fixed, and methionine oxidation as a variable modification. Precursor mass tolerance was set to 5 ppm, fragment ion mass tolerance was set to 0.01 Da, the maximum allowed variable PTMs and maximum number of missed cleavages were set to 5. Non-specific cleavages were allowed at one end of the peptide. The cleavage site for each enzyme were at the C-terminus of: R/K for trypsin, R/K/D/E for LysC/Trypsin, F/L/M/W/Y for chymotrypsin, K/D/E for GluC/LysC, and A/C/G/M/F/S/Y/W for proteinase K.

MeroX Data Analysis

Photocrosslinking experiments were analysed using MeroX 1.6.6. The search database contained the VSD2-NavAb sequence and the respective HTMp probe sequence. For each enzyme predicted cleavage site, three missed cleavages were allowed and the option for semi-unspecific digestion was selected. Carbamidomethylation was set as a fixed modification and represented as B. Methionine oxidation (m) was set as variable modification with a maximum of two occurrences per peptide. The photoprobe sequence in the provided database contained L-photomethionine represented by p. In the search, the crosslinker was set as p ($C_6H_9N_3O$) requiring loss of N_2 and all amino acids were considered as potential crosslinking sites. Modification of fragments of the photomethionine-containing peptide was set to -28.0061 for loss of N_2 and -9.9956 for loss of N_2 and addition of H_2O. The precursor and fragment ion tolerance was 10 ppm and the FDR cut-off for reported spectra was set to 0.05. The peptide crosslink identifications that were considered as significant always had a higher score than the decoy hits.

QUANTIFICATION AND STATISTICAL ANALYSIS

All Nav channel inhibitions were presented as mean ± standard error of the mean from at least three independent trials. IC_{50} values were calculated by using Prism (version 6.05, Graphpad, CA) four-parameter Hill equation fitting of the dose-response curve, with each point $n = 3 \sim 20$.

DATA AND CODE AVAILABILITY

The accession number for the raw MS data reported in this paper is ProteomeXchange Consortium: PXD015037. The raw MS data have been deposited to the ProteomeXchange Consortium (http://proteomecentral.proteomexchange.org) via the PRIDE partner repository PXD015037.

Cell Chemical Biology, Volume *27*

Supplemental Information

Development of Photocrosslinking Probes Based on Huwentoxin-IV to Map the Site of Interaction on Nav1.7

Foteini Tzakoniati, Hui Xu, Tianbo Li, Natalie Garcia, Christine Kugel, Jian Payandeh, Christopher M. Koth, and Edward W. Tate

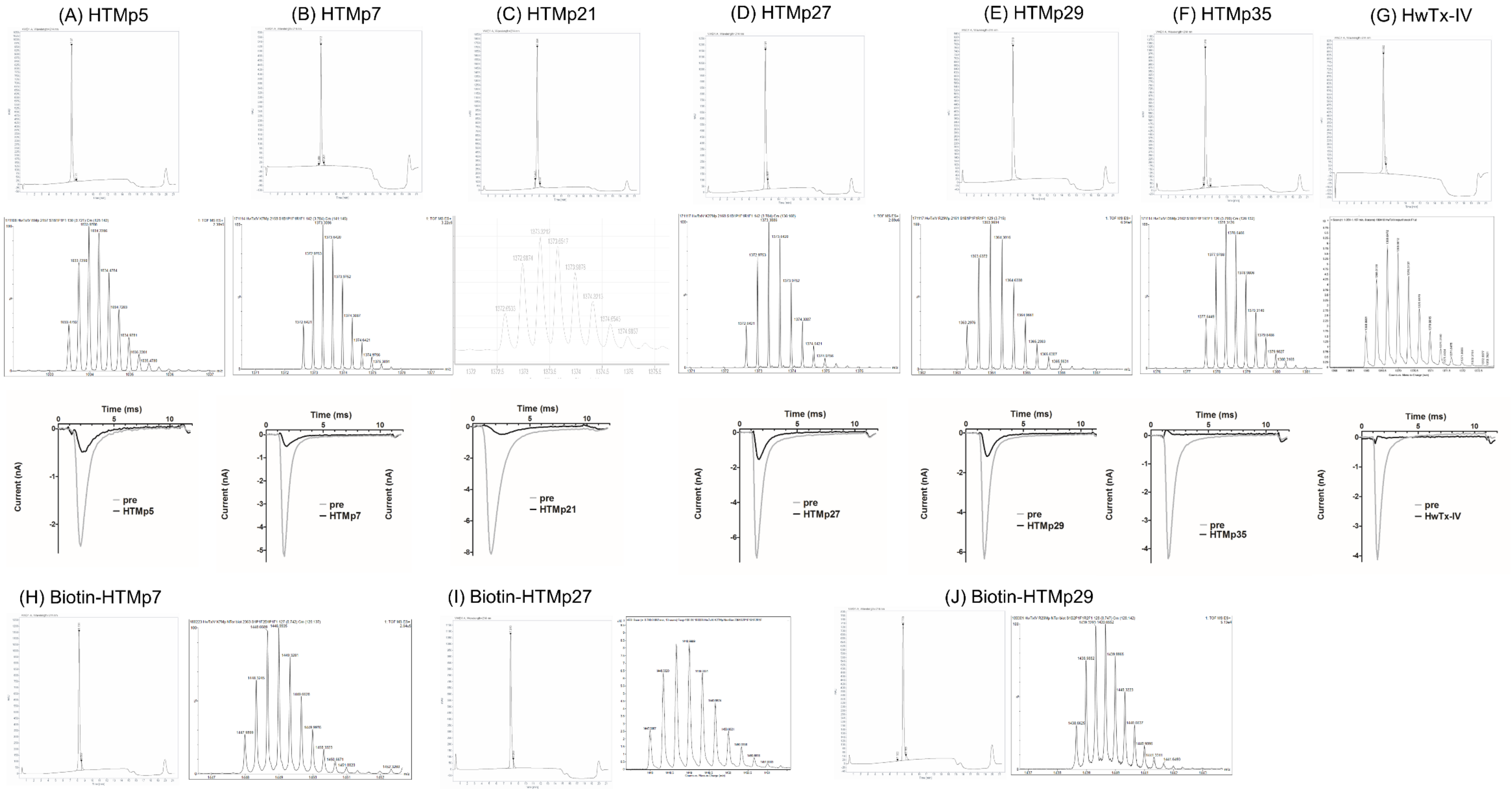

Figure S1. Related to Figure 1. LCMS traces of peptides and effects at 200 nM on CHO cells expressing Nav1.7

(A) HTMp5 (B) HTMp7 (C) HTMp21 (D) HTMp27 (E) HTMp29 (F) HTMp35 (G) HwTx-IV (H) biotin-HTMp7 (I) biotin-HTMp27 (J) biotin-HTMp29

Figure S2. Related to Figure 3. Competition-depended photocrosslinking

(A) FLAG purification stain-free SDS-PAGE. The purified channel was collected in four elutions using the FLAG peptide. (B) Size-exclusion elution profile. Extraction from insect cell membranes and further purification steps required the use of GDN detergent. The fractions were pooled to obtain a homogeneous sample of the tetrameric channel (blue bar). (C) Stain-free SDS-PAGE of VSD2-NavAb after size-exclusion chromatography. The blue bar indicates the fractions that were pooled for photocrosslinking experiments. (D) HTMp29 photocrosslinking. Competition with increasing concentration of HwTx-IV and stain-free SDS-PAGE analysis of molecular weight shift. (E) Biotin-HTMp photoprobes photocrosslinking. Competition with HwTx-IV (150 μM) and stain-free SDS-PAGE analysis of molecular weight shift.

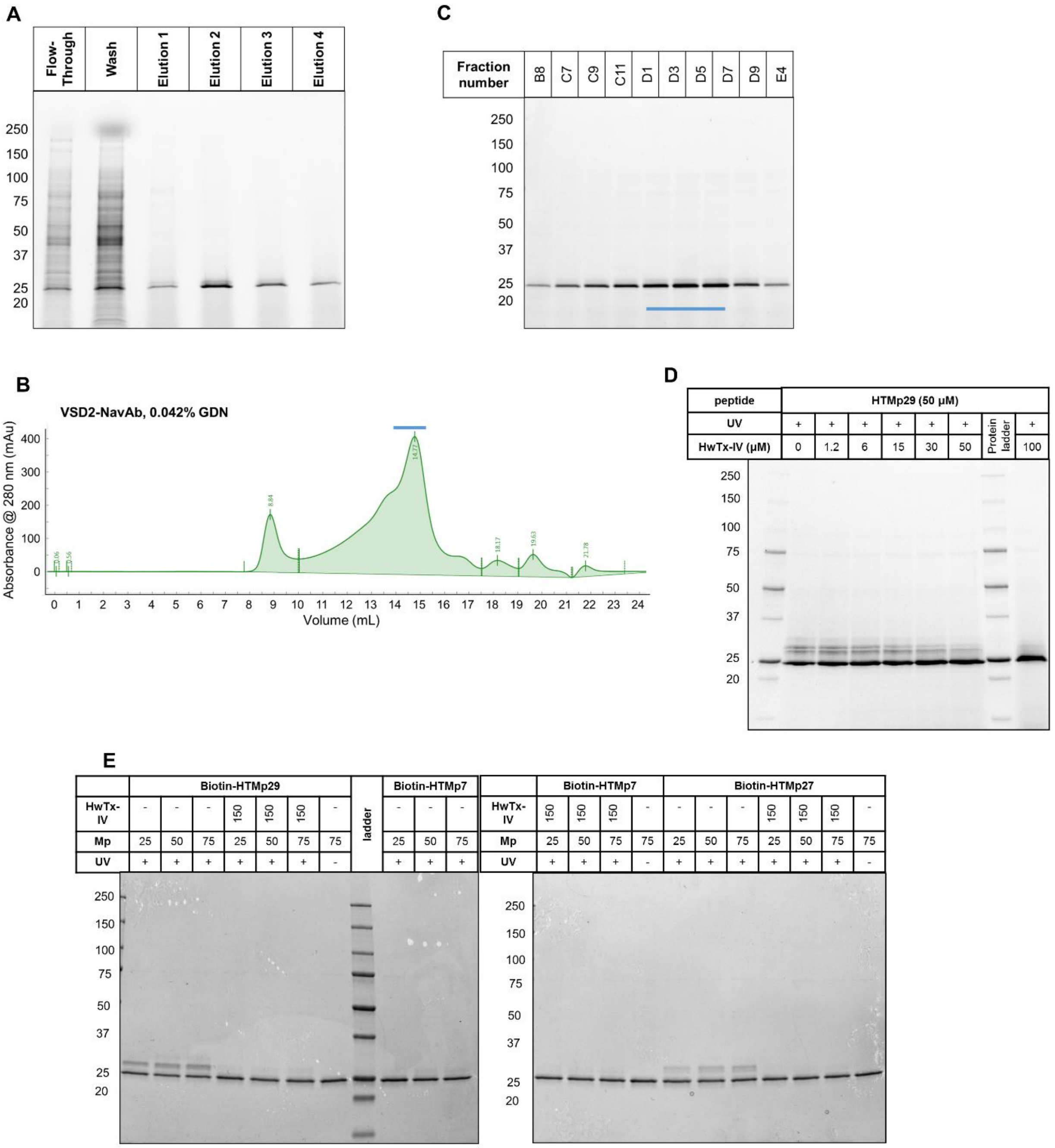

Figure S3. Related to Figure 4. MS spectra of crosslinked peptides

Mp27 photocrosslinking, proteinase K

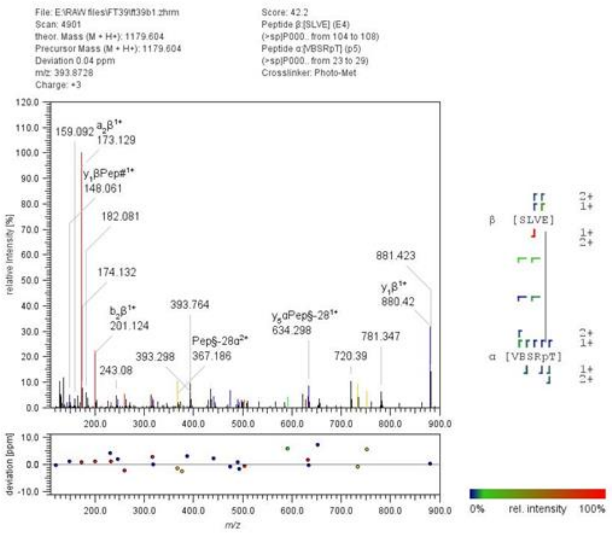

Mp27 photocrosslinking, proteinase K

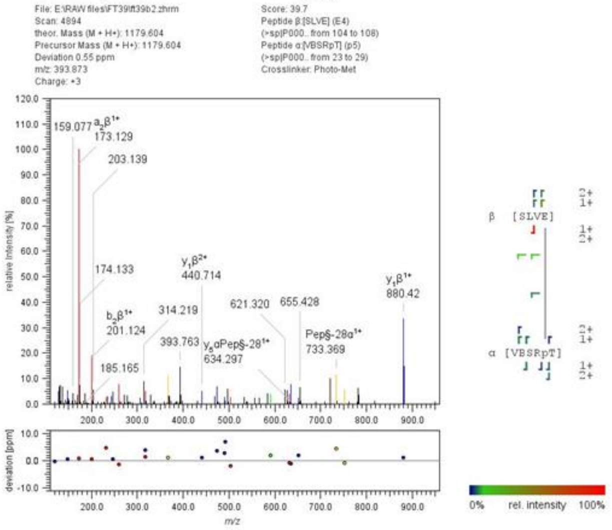

Mp29 photocrosslinking, proteinase K

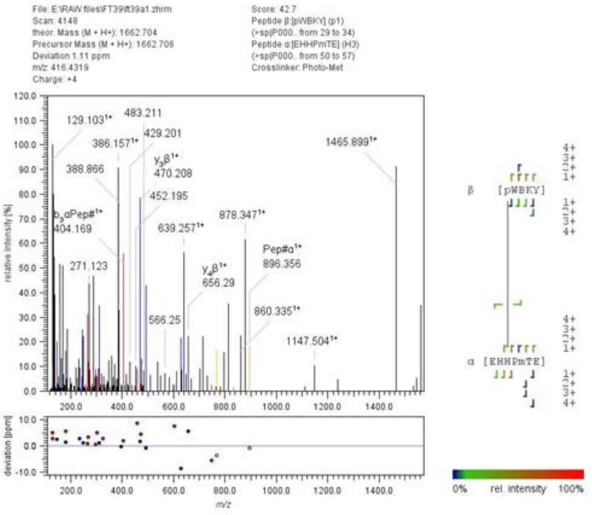

Mp29 photocrosslinking, proteinase K

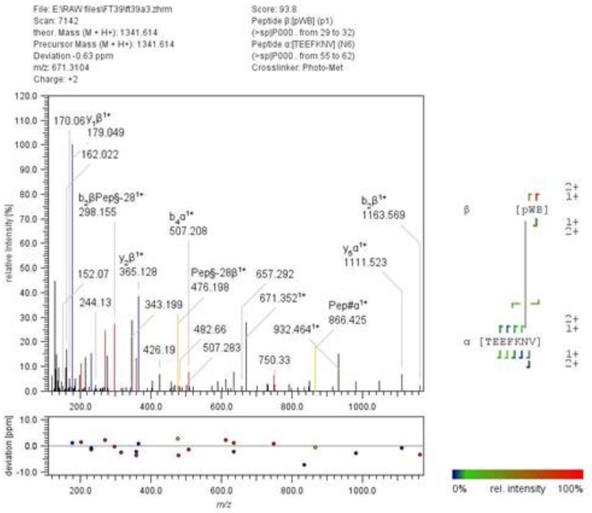

Mp29 photocrosslinking, proteinase K

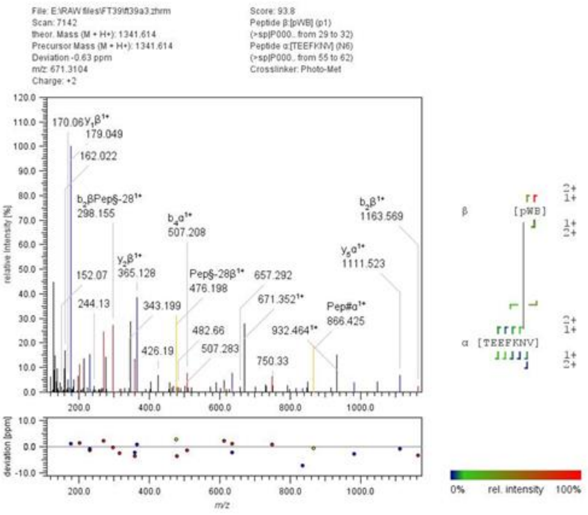

Mp29 photocrosslinking, trypsin

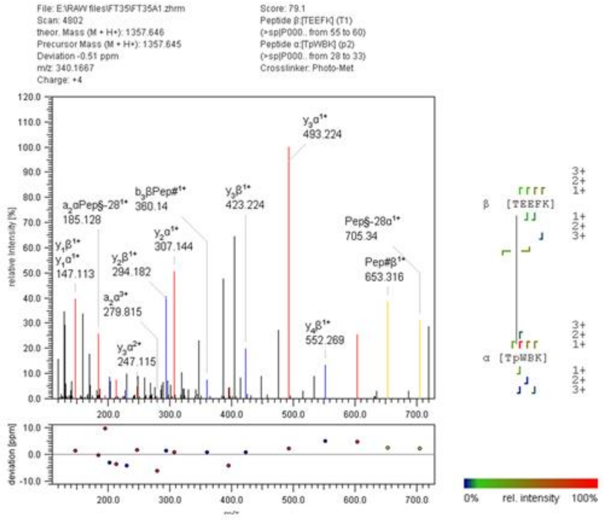

Figure S4. Related to Figure 4. Sequence coverage of VSD2-NavAb with different digestion enzymes

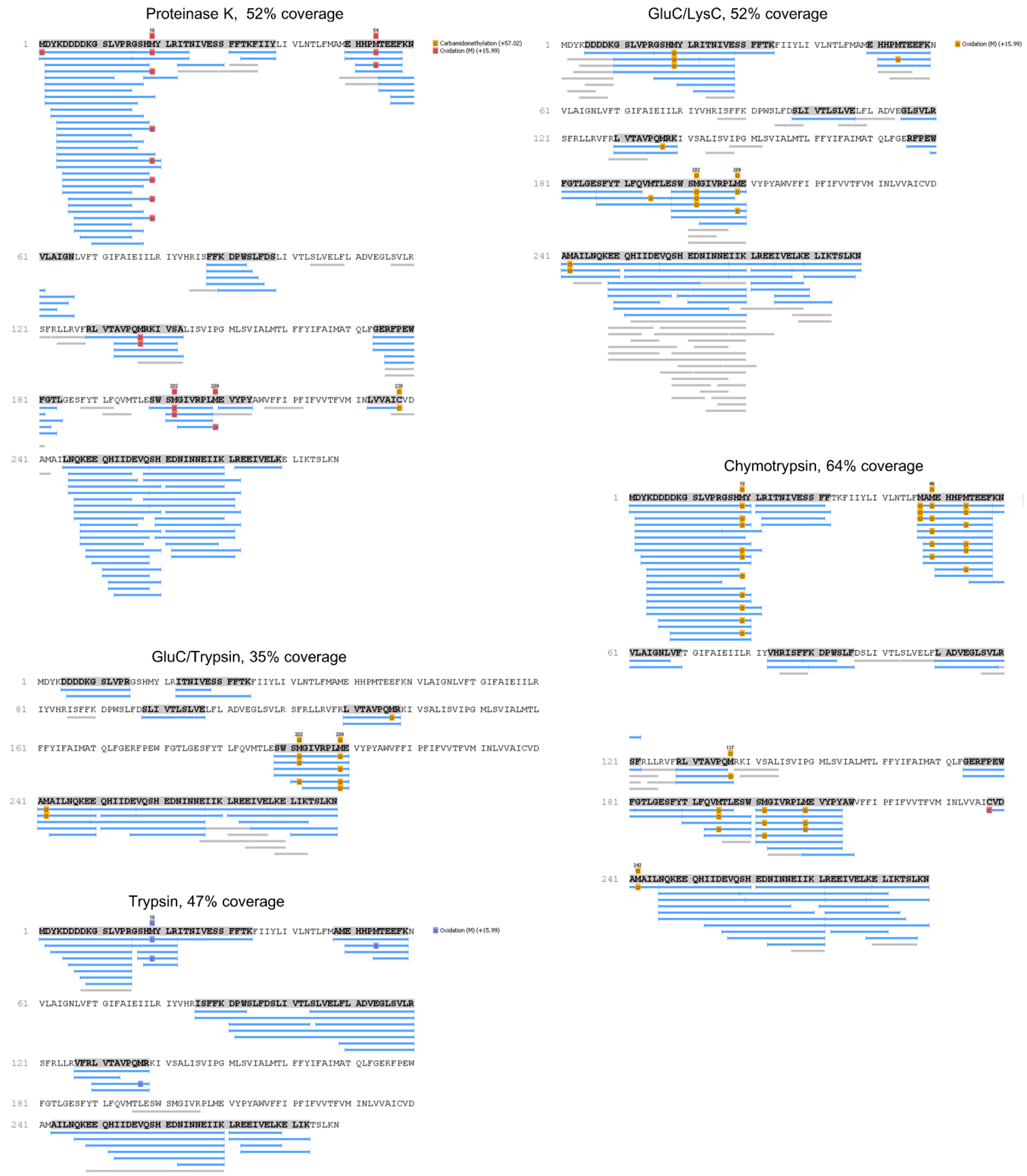

Supplement: Document S2. Article plus Supplemental Information [file mmc2.pdf]
